# Obesity surgery improves metabolic dysfunction-associated steatotic liver disease and type 2 diabetes – MRI and biochemical analysis of liver and pancreas

**DOI:** 10.1016/j.sopen.2025.12.006

**Published:** 2026-01-05

**Authors:** Hannes Götz Kenngott, Philipp Anthony Wise, Yixin Jiang, Amila Cizmic, Felix Wagner, Hans-Ulrich Kauczor, Adrian T. Billeter, Lars Fischer, Johanna Nattenmüller, Beat Peter Müller-Stich, Rainer Grotelüschen, Felix Nickel

**Affiliations:** a Department of General, Visceral, and Transplantation Surgery, Heidelberg University Hospital, Im Neuenheimer Feld 110, 69120, Heidelberg, Germany; b Department of Diagnostic and Interventional Radiology, Heidelberg University Hospital, Im Neuenheimer Feld 110, 69120, Heidelberg, Germany; c Department of Abdominal Surgery, Clarunis – Academic Centre of Gastrointestinal Diseases, St. Clara and University Hospital of Basel, Petersgraben 4, 4051, Basel, Switzerland; d Department of Surgery, Hospital Mittelbaden, Balgerstrasse 50, 76532, Baden-Baden, Germany; e Department of Diagnostic and Interventional Radiology, Medical Center - University of Freiburg, Faculty of Medicine, University of Freiburg, Freiburg, Germany; f Department of General, Visceral, and Thoracic Surgery, University Medical Center Hamburg-Eppendorf, Martinistrasse 52, 20246, Hamburg, Germany

**Keywords:** Obesity surgery, MASLD, Type 2 diabetes mellitus, Fatty pancreas, MRI, Image processing

## Abstract

**Background:**

This study evaluated changes in metabolic dysfunction-associated steatotic liver disease (MASLD), type 2 diabetes mellitus, liver volume, liver/pancreas fat in patients after obesity surgery.

**Methods:**

Magnetic Resonance Imaging (MRI) measured liver volume/fat and pancreas fat in 31 patients with laparoscopic sleeve gastrectomy (LSG, *N* = 20) or Roux-en-Y gastric bypass (RYGB, *N* = 11) preoperatively and at 3- and 12-month follow-up. Clinical data and blood values were taken concomitantly to calculate Non-alcoholic fatty liver disease (NAFLD) score.

**Results:**

The percentage total weight lost (17.5 % ± 5.4 % at 3 months, 28.4 % ± 8.3 % at 12 months) and percentage excess weight lost (40.0 % ± 11.8 % at 3 months, 65.0 % ± 18.8 % at 12 months) were significant. Liver volume decreased from 2378.3 ± 514.5 cm^3^ to 1928.7 ± 333.5 cm^3^ at 3 months (*p* < 0.001) and 1685.0 ± 310.9 cm^3^ at 12 months (*p* < 0.001) after surgery. Liver fat percentage decreased from 16.7 % ± 10.3 % to 8.7 % ± 5.4 % at 3 months (p < 0.001) and 5.2 % ± 3.6 % at 12 months (p < 0.001). Pancreatic fat percentage showed a reduction from 14.8 % ± 5.5 % to 10.9 % ± 4.9 % at 3 months (*p* = 0.007) postoperatively. NAFLD score improved from preoperative measurements to 12 months postoperatively (−0.89 ± 1.54 vs. -1.77 ± 1.25, *p* < 0.019). Preoperatively, 22 of 31 (71 %) patients had advanced/intermediate scores; 12 months postoperatively only 12 (39 %) remained (*p* = 0.044). No significant differences between LSG and RYGB were found regarding goal parameters.

**Conclusion:**

Obesity surgery reduced liver volume, type 2 diabetes, fat content of liver and pancreas and improved indicators of MASLD. No significant difference in outcome between operation methods could be established.

## Introduction

Obesity is a significant and increasing problem in healthcare worldwide. An increasing proportion of both the adult and paediatric population are overweight or obese [[1], [2], [3]], and current evidence projects that this trend will continue for the foreseeable future [2]. In addition, the development of other significant healthcare problems is associated with obesity or metabolic syndrome, among these varying types of cancer, atherosclerosis, type 2 diabetes mellitus, hypertension, gastroesophageal reflux disease, and metabolic dysfunction-associated steatotic liver disease (MASLD) [[3], [4], [5], [6], [7], [8], [9], [10], [11]]. Of particular interest to this study is MASLD, which has an increasing incidence and is associated with an increased risk of cirrhosis, hepatocellular carcinoma, and liver failure [4,[12], [13], [14]]. MASLD represents a progressive spectrum of liver disease that can advance from simple steatosis to steatohepatitis, fibrosis, and ultimately cirrhosis. The progression to cirrhosis is a major concern as it significantly increases the risk of developing hepatocellular carcinoma. Furthermore, the development of cirrhosis in MASLD patients is associated with increased liver-related mortality, with hepatocellular carcinoma and liver failure representing major causes of death in this population [3,8,9]. In this context, Obesity surgery has become an increasingly important treatment option for overweight patients [1,4,[15], [16], [17], [18], [19]], addressing effectively both the issue of overweight and comorbidities associated with metabolic syndrome. Measuring post-surgical changes to liver volume and parameters indicative of MASLD (i.e., fibrosis score), is therefore of clinical interest. Additionally, non-alcoholic fatty pancreas disease (NAFPD) is a growing global health concern that has been shown to be associated with type 2 diabetes and obesity [20]. NAFPD is characterized by the accumulation of excessive fat within the pancreas, which has recently been associated with insulin resistance and decreased insulin secretion [21,22]. The present study aimed to assess outcome of patients after Roux-en-Y gastric bypass and laparoscopic sleeve gastrectomy with regards to weight loss, changes in liver volume, liver fat percentage and pancreatic fat percentage and the correlations of these parameters to the clinical endpoints of active type 2 diabetes mellitus and Non-alcoholic fatty liver disease (NAFLD) fibrosis score.

## Methods

The present study is part of a prospective longitudinal cohort study begun in 2011 at the Department of General, Visceral, and Transplantation Surgery at Heidelberg University Hospital evaluating *Postoperative Constitutional Changes* in *Obesity* (POCCO) [23]. The main goal of the POCCO study was the evaluation of changes in gastric size after obesity surgery in patients with a preoperative BMI >35 kg/m^2^ with respect to weight regain after surgery. The present study is an evaluation of the liver and pancreas segmentations taken from the whole-body MRI data of POCCO patients [23] in conjunction with analysis of NAFLD fibrosis scores, type 2 diabetes and clinical outcomes in the patient population.

### Patients

Patient criteria for inclusion into the study were a BMI between 35 and 60 kg/m^2^ and an indication for obesity surgery according to German S3 guidelines as evaluated in a multidisciplinary setting in an obesity centre. A psychosomatic and metabolic profile was performed on all patients to ensure no contraindications to surgery were present, as well as screening for common comorbidities of metabolic syndrome: Type 2 diabetes mellitus, arterial hypertension, sleep apnoea, and gastroesophageal reflux (GERD). Changes to the comorbidities in this population were presented in the previous 2019 publication along with changes in body composition (visceral/subcutaneous adipose tissue and skeletal muscle) [23]. Criteria for exclusion were pregnancy, inability to consent or inability to receive an MRI. MRIs for the POCCO study were performed between July 2012 and March 2017 before surgery, and again at 3- and 12-months post-surgery for each participant. Standard clinical postoperative assessment was additionally performed at four weeks and six months after surgery to exclude surgical complications such as stenosis, adhesions or hernia. Patients were followed up on their diet, exercise and activity, and psychological well-being at the postoperative measurement intervals. Routine assessment and consultation with a dietician were arranged on an as-needed basis.

### Magnetic resonance imaging

MRI scans were performed at the Department of Diagnostic and Interventional Radiology of Heidelberg University Hospital using a “MAGNETOM Aera” 1.5 Tesla MRI-scanner with a 70 cm wide bore design (Siemens Healthcare GmBH, Erlangen, Germany). A 2-point Dixon protocol with gradient echo technique using parallel acquisition mode for high-resolution images (T1 Dixon vibe, 3 mm slice thickness, 1.302 × 1.302 mm matrix) was chosen. No contrast agent was used. MR-software, hardware and protocol were constant across the study measurements.

### Image segmentation with the medical imaging interaction toolkit (MITK)

**Fig. 1:**
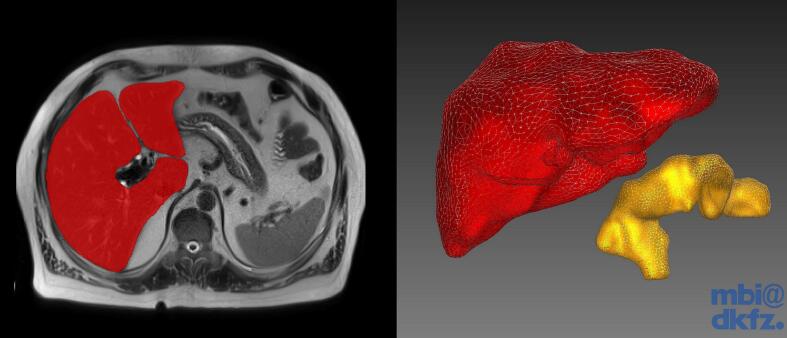
Medical Imaging interaction toolkit (German Cancer Research Center (DKFZ), Heidelberg, Germany) segmentations of liver (red) and pancreas (yellow). (For interpretation of the references to colour in this figure legend, the reader is referred to the web version of this article.)

MRI Data was processed and segmented using the Medical Imaging Interaction Toolkit (MITK) [24], an open source image processing software, based on the open source tools Visualization Toolkit (VTK) (Kitware Inc., Clifton Park, New York, USA), Insight Segmentation and Registration Toolkit (ITK) (United States National Library of Medicine, Bethesda, Maryland, USA; Kitware Inc., Clifton Park, New York, USA; et al.), and Qt Bibliothek (The Qt Company, Espoo, Finland) (Fig. 1).

### Fat percentage calculation

The liver and pancreatic fat percentages were analysed using OsiriX image post-processing software (OsiriX, Pixmeo SARL, Bernex, Switzerland) [25], which allows the estimation of tissue intensity using a predefined calculation area, or region of interest (ROI). Three ROIs were placed in liver segments 4, 7 and 8, and 3 ROIs were placed in the head, body, and tail of the pancreas [26]. Care was taken to place all ROIs in the middle of organ parenchyma, avoiding vessels, ducts, cysts, and adjacent fat. These identical ROIs were copied in identical areas within the fat and water image derived from the 2-point Dixon sequence. The resulting mean of all three ROIs per organ was calculated on an individual patient basis, and the percentage fat content was then calculated using the formula

$$\mathrm{fat}\;\mathrm{fraction}\;\left[\%\right]=\frac{\mathrm{mean}\;{\mathrm{intensity}}_{\mathrm{fat}\;\mathrm{image}}}{\mathrm{mean}\;{\mathrm{intensity}}_{\mathrm{fat}\;\mathrm{image}}+\mathrm{mean}\;{\mathrm{intensity}}_{\mathrm{water image}}}.$$

This method was previously used by Bertheau et al. [27] in their study for calculating bone marrow fat content using MRI scans.

### Laboratory values and NAFLD fibrosis scoring system

Laboratory parameters to calculate fibrosis scores as well as blood lipid levels were assessed preoperatively as well as three and twelve months postoperatively. The liver biomarkers of AST, ALT, albumin and platelet count were analysed and graded according to the NAFLD fibrosis score, chosen for its relevant use and validity in current obesity surgery and MASLD literature [28,29]. Blood lipid levels were assessed for average changes across the twelve-month period, as well as changes to the total cholesterol to HDL ratio. The presence or absence of Type 2 diabetes in the study population was defined as the individual requiring antidiabetic medication or insulin at the point of measurement.

### Statistics

The results of the image segmentations and laboratory measurements were collected in Microsoft Excel 2016, the statistical analysis was carried out with IBM SPSS Statistics 24 and Python 3.6 with pandas and scipy packages. Figure graphics were created using Python 3.6 with pandas and seaborn packages. Related data samples were compared using a two-tailed *t*-test assuming unequal variances. Correlation between two independent variables was calculated using Spearman's r. Correlation between surgical intervention and presence or absence of a high fibrosis score was evaluated using a chi-squared test. Statistical significance for all tests was set at *p* ≤ 0.05.

## Results

Patients (18 women and 13 men) received RYGB [11] or LSG [20]. At surgery, patients had an age of 46.4 ± 9.7 years, and BMI of 45.6 ± 6.3 Kg/m^2^ (Table 1). Over the study, three patients (9.7 %) were lost to follow-up, resulting in a final study number of *N* = 28. The results only include the patients who completed the study until and including the 12-month follow-up. Patients had significant postoperative weight loss and reduction in BMI [23].

**Table 1:** Total study average of body changes over time, changes in blood lipid levels, cholesterol to HDL ratio, levels of AST, ALT and ratio of AST/ALT compared to initial measurement. Weight lost given as change relative to weight at 0 months [%]. %TWL = percent total weight lost; %EWL = percent excess weight lost. p^1^: Value compared to initial preoperative measurement. p^2^: Value compared to measurement at three months. (*): cited from Kenngott et al., 2019 [23].

Total study (*N* = 28)						
	Initial Values	3 Months		12 Months		
	Value	Value	p^1^	Value	p^1^	p^2^
BMI^(⁎)^	45.6 ± 6.3	37.2 ± 5.6	**<0.001**	32.2 ± 5.3	**<0.001**	**<0.001**
Weight [kg] ^(⁎)^	137.1 ± 19.2	112.4 ± 17.4	**<0.001**	97.2 ± 16.5	**<0.001**	**<0.001**
%TWL [%]^(⁎)^		17.5 ± 5.4		28.4 ± 8.3		
%EWL [%]^(⁎)^		40.0 ± 11.8		65.0 ± 18.8		
Type 2 Diabetes [n]	15	9	0.09	5	**0.007**	0.29
Chol [mg/dl]	181.7 ± 36.2	173.6 ± 34.4	0.376	185.9 ± 38.1	0.666	0.19
HDL [mg/dl]	41.5 ± 8.3	41.8 ± 7.0	0.853	54.6 ± 11.3	**<0.001**	**<0.001**
LDL [mg/dl]	105.6 ± 30.4	104.4 ± 30.7	0.881	108.3 ± 31.4	0.732	0.62
Chol/HDL	4.5 ± 1.0	4.2 ± 1.0	0.32	3.5 ± 0.8	**<0.001**	**0.002**
AST [U/l]	25.3 ± 9.6	22.1 ± 12.3	0.266	19.8 ± 8.0	**0.019**	0.15
ALT [U/l]	39.6 ± 19.8	28.5 ± 20.0	**0.036**	22.1 ± 13.5	**<0.001**	0.39
AST/ALT	0.7 ± 0.4	0.9 ± 0.4	**0.09**	1.1 ± 0.5	**0.006**	0.19

### Liver volume

**Fig. 2:**
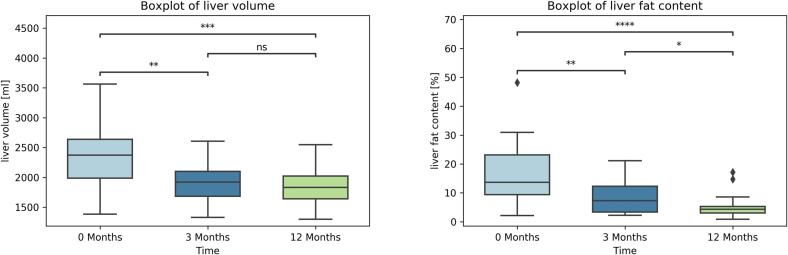
Boxplot of average liver volume and boxplot of average liver fat content at measurement times. Outliers marked as a diamond. *p*-value annotation legend: **: *p* < 0.01, ***: *p* < 0.001, ns: not significant.

Liver volume decreased significantly three and twelve months after surgery compared to preoperative volume. Volume loss was higher over the first three months than over the following nine months. When comparing surgical procedures, no significant difference between pre-op volumes, changes after three months, or changes after twelve months was found between LSG and RYGB. Results are summarized in Fig. 2 and in Table 2.

**Table 2:** Comparison of Liver Volume, liver fat percentage, pancreas fat percentage, and MASLD fibrosis score by surgical procedure (RYGB vs LSG). Comparison showed no significant differences in analysed parameters.

Comparison by surgery		Preoperative value	3 Months	12 Months
liver volume [ml]	RYGB (*n* = 10)	2439 ± 394	2027 ± 283	1941 ± 267
	LSG (*n* = 18)	2347 ± 584	1871 ± 357	1827 ± 330
	p	0.612	0.207	0.323
liver fat [%]	RYGB (n = 10)	15.6 ± 9.1	7.4 ± 4.8	4.1 ± 1.9
	LSG (n = 18)	17.3 ± 11.0	9.4 ± 5.7	5.7 ± 4.1
	p	0.662	0.335	0.151
pancreas fat [%]	RYGB (n = 10)	13.8 ± 5.6	12.6 ± 6.1	11.1 ± 5.5
	LSG (n = 18)	15.4 ± 5.6	12.7 ± 6.3	10.8 ± 4.7
	p	0.489	0.963	0.912
MASLD fibrosis score	RYGB (n = 10)	−1.17 ± 1.03	−1.6 ± 0.84	−1.92 ± 0.91
	LSG (*n* = 18)	−0.8 ± 1.76	−1.4 ± 1.6	−1.69 ± 1.4
	p	0.596	0.672	0.597

### Liver and pancreas fat content

**Fig. 3:**
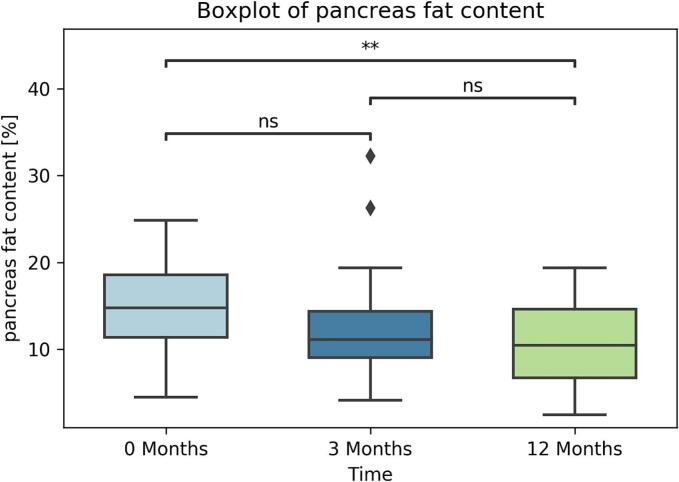
Boxplot of average pancreatic fat content at measurement times. Outliers marked as a diamond. p-value annotation legend: **: p < 0.01, ns: not significant.

The liver and pancreatic fat content decreased significantly on average across the patient population. Liver fat percentage decreased at three months (*p* < 0.001) and twelve months (p < 0.001 compared to initial, *p* = 0.002 compared to three-month values) postoperatively. Pancreatic fat percentage showed a non-significant reduction from initial measurement to three months (*p* = 0.08) and showed a significant reduction at twelve months postoperatively (*p* = 0.007 compared to initial, *p* = 0.23 compared to three-month values). Results are summarized in Fig. 2, Fig. 3.

### NAFLD fibrosis score

**Fig. 4:**
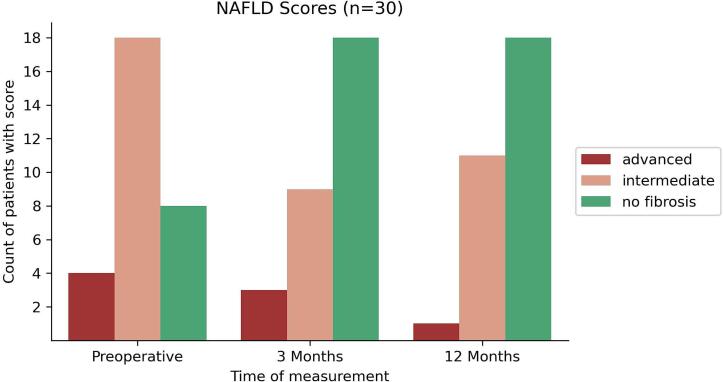
NAFLD fibrosis score by patient. Cut-off for advanced fibrosis is <0.676, cut-off for intermediate fibrosis is < −1.455. The chi-square statistic (test of independence) for determining effect across the population is 9.8218. The p-value is 0.044. The result is significant at *p* < 0.05.

The NAFLD fibrosis score showed a significant improvement at twelve months after obesity surgery. At baseline, the average NAFLD score was −0.89 ± 1.54. This value is indicative of intermediate fibrosis according to the METAVIR grading system. After three months, average NAFLD score was −1.47 ± 1.38 (*p* = 0.131 compared to preoperative baseline) and after twelve months −1.77 ± 1.25 (*p* = 0.019 compared to preoperative baseline and *p* = 0.39 compared to three months postoperatively). Both values are indicative of no fibrosis according to the METAVIR grading system [30]. Results are summarized in Table 2. When comparing individual results of the scoring system across the patient population, the number of patients with advanced or intermediate fibrosis according to NAFLD fibrosis score decreased after three months and 12 months. A Chi-squared test of independence was performed to examine the relationship of NAFLD fibrosis score results before and after surgery and showed a statistically significant difference between pre-and postoperative scores. Results are summarized in Fig. 4.

### Type 2 diabetes mellitus

The presence of clinically relevant type 2 diabetes mellitus was evaluated preoperatively and on follow-up, defined as whether patients actively required other antidiabetic medication or insulin. There was a significant reduction of patients actively taking oral antidiabetics or insulin at 12 months postoperatively compared to preoperative count (*p* = 0.007). Results are summarized in Table 1.

### Potential influencing factors for outcome

**Fig. 5:**
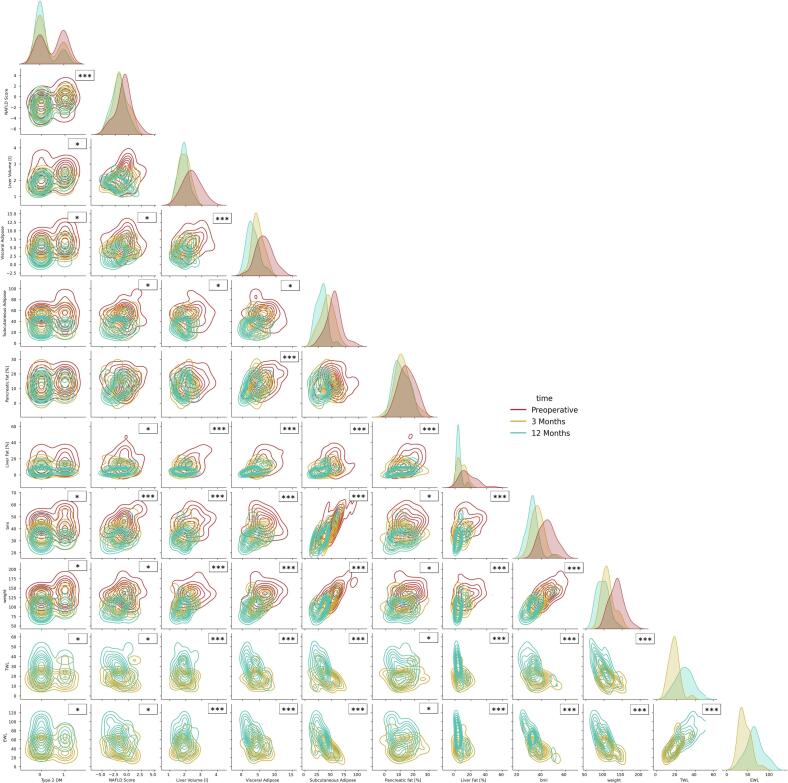
Kernel density estimate plot of outcome variable correlations at differing measurement times. The diagonal plot shows the individual variable kernel density estimates. p-value annotation legend: *: p < 0.05, ***: *p* < 0.001.

All findings and significant correlations between the outcome parameters are visualized in Fig. 5 as a Kernel Density Estimate (KDE) plot. KDE plots visualize probability density functions by using Gaussian functions over data points. Fig. 5 shows relationships between variables before and after surgery. Diagonal elements represent individual variables, while off-diagonal elements show bivariate relationships. Correlation strength and direction can be determined from the KDE plots. When comparing the outcome parameters across the patient population, strong correlations were found between visceral adipose tissue and the remaining parameters (type 2 diabetes, liver volume, NAFLD score, subcutaneous adipose tissue, pancreas fat percentage, and liver fat percentage), as well as between liver fat percentage and these parameters (type 2 diabetes, liver volume, NAFLD score, subcutaneous adipose tissue, pancreas fat percentage, and liver fat percentage). Significant correlations were also observed between NAFLD score and most remaining parameters (type 2 diabetes, liver volume, NAFLD score, subcutaneous adipose tissue, and liver fat percentage), except for pancreas fat percentage.

### Lipids

Cholesterol/HDL ratio significantly decreased twelve months after surgery. This change in the ratio resulted from a significant increase in HDL levels at twelve months. Both total cholesterol and LDL levels showed no significant changes between measurements. Results are summarized in Table 1.

### ALT/AST ratio

ALT/AST ratio significantly increased twelve months after surgery. Both AST and ALT levels were significantly reduced after twelve months. The change in the AST/ALT ratio resulted from a greater reduction in ALT levels than AST levels, both at three and at twelve months. Results are summarized in Table 1.

### Comparison of surgical procedures

When comparing patients who had undergone LSG to patients who had undergone RYGB, no significant differences in liver volume, liver or pancreatic fat percentage, or NAFLD fibrosis score were measured at any of the measurement points. Patients from both groups had comparably similar liver volumes preoperatively (*p* = 0.61) as well as at 3 months (*p* = 0.21) and 12 months (*p* = 0.32). Patients from both groups had comparably similar liver fat content preoperatively (*p* = 0.66) as well as at 3 months (*p* = 0.34) and 12 months (*p* = 0.15). Patients from both groups had comparably similar pancreatic fat content preoperatively (*p* = 0.49) as well as at 3 months (*p* = 0.96) and 12 months (*p* = 0.91). Patients from both groups had comparable NAFLD fibrosis scores preoperatively (p = 0.6) as well as at 3 months (*p* = 0.67) and 12 months (p = 0.6). Results are summarized in Table 2.

## Discussion

The current study showed a significant reduction in liver volume and liver fat content following obesity surgery after three and twelve months. Pancreas fat content and the average NAFLD fibrosis score showed a significant improvement at twelve months compared to preoperatively. In addition, the number of patients categorized with either advanced or intermediate fibrosis according to the METAVIR grading system also decreased after surgery [30]. No significant differences were found between RYGB and LSG.

Over the study population, both liver and pancreatic fat content decreased significantly after surgery, and a significant correlation between both values could be established. This is in keeping with the current literature showing a reduction of visceral adipose tissue after obesity surgery [12,19,23]. Additionally, a relation between fatty liver and fatty pancreas has been shown previously as well as relations between type 2 diabetes mellitus and MASLD [4]. The percentage fat content in organ parenchyma has also been shown to correlate with metabolic health [31,32], further strengthening the argument that obesity surgery can aid in improving patient health beyond weight reduction. The current study showed no significant correlation between the percent concentration of pancreatic fat and type 2 diabetes, which is in contrast to previous studies that have shown a strong association between pancreatic fat and insulin resistance [33]. This effect might however be detected with higher sample sizes. The current study results also suggest that other factors, such as total visceral adipose tissue, may also have an important relationship with type 2 diabetes. Previous studies have also shown that weight loss through obesity surgery can reduce the incidence of type 2 diabetes [34,35]. In particular, RYGB has been shown to significantly improve insulin sensitivity and glucose metabolism. These findings suggest that the reduction in total body weight or total body fat content after obesity surgery may play a more important role in the improvement of insulin sensitivity and glucose metabolism than the reduction of pancreatic fat percentage.

In the current study, the most marked change to liver volume happened in the first three months following surgery. Additionally, a high correlation between liver fat percentage and both SAT and VAT was measurable in the patient population. It is therefore likely that the reduction in liver fat percentage correlated with reduction in total adipose tissue after surgery, which was also more pronounced in the first three months. Of note is also the high correlation between liver volume and visceral adipose tissue. In other studies, correlations between body weight and liver health have been firmly established [4,[36], [37], [38]]. Liver volume and liver health have been negatively correlated in both volume extremes, both in the case of significantly increased liver volume, such as in MASLD [39], as well as significantly reduced liver volume, such as in small for size syndrome after liver resections [40]. Additionally, lean liver volume appears to be a better indicator of liver metabolic function than total liver volume [41], and lean liver volume has been found to correlate more closely with lean body mass and gender than total body weight [42]. In the current study, the most likely cause of rapid volume reduction appears to have been a decrease in fat content of liver tissue, as significant correlation between liver fat and liver volume was established. This would also be in keeping with the reduction in both subcutaneous and visceral adipose tissue described in this patient population [23]. Furthermore, the improvement in average MASLD fibrosis score, AST/ALT ratio and lipid panel indicate that the reduced liver volume is a marker of desired physiological change, i.e., loss of liver fat and not lean liver volume as a result of the operation, rather than a pathologic process. The decrease in visceral adipose tissue as observed in the previous publication on the current study population, as well as changes to liver volume in other studies researching weight loss after obesity surgery provide additional basis for this argument [12,19,23,36,43,44]. This is also in keeping with the results from a recent study by Chiyanika et al., which found significant causal relationships between steatohepatosis and obesity in their study population [38]. The reduction in NAFLD score and improvement in plasma lipid profile in the above measurements provide additional support for this explanation.

In the results of the current study, the average NAFLD fibrosis score decreased significantly after surgery. The number of patients categorized as having advanced fibrosis according to the clinically validated METAVIR grading system decreased after three months, and remained decreased after twelve months. This further supports the hypothesis that obesity surgery improves metabolic health, as the NAFLD fibrosis score has shown high clinical sensitivity and specificity in diagnosing advanced liver fibrosis [28,29]. This is also in line with existing literature assessing liver health and fibrosis post obesity surgery using scoring systems [45], diagnostic tools such as elastography measurements [12], voxel-wise analysis [46] and histological biopsy analysis. When examining the factors that make up the NAFLD score in particular, the major changes leading to the reduction in scores was the reduction of clinically significant type 2 diabetes mellitus in the study population [23], as well as the ALT/AST ratio. The ratio of ALT to AST increased significantly over the follow-up period in the present study, while the total levels of both AST and ALT significantly decreased after twelve months, ALT decreasing at a greater percentage than AST. These results confirm previous findings of both absolute reduction in ALT, as well as the ALT/AST ratio increase after surgery [47,48].

A direct comparison between the two procedures used, RYGB and LSG, could not find differences in either liver volume, liver or pancreatic fat percentage, or NAFLD fibrosis score at any measurement point. As the overall study did show improvements to all these parameters over 12 months, this finding may indicate that RYGB and LSG have comparable efficacy regarding improving liver function. Our findings align with a recent meta-analysis by Baldwin et al., which reported similar improvements in hepatic steatosis and fibrosis markers between LSG and RYGB at 12-month follow-up, as well as a large comparative study by Cherla et al. which included 489 patients, showing similar efficacy for both procedures [49,50]. Both procedures appear to achieve comparable weight loss and metabolic improvements in the first postoperative year, which may explain the similar hepatic outcomes observed.

Over the 12-month measurement period, a significant increase in HDL was observed. Both LDL and total cholesterol levels did not significantly decrease or increase. This finding may suggest a prolonged metabolic change in response to obesity surgery. This change to HDL levels resulted in a reduced total cholesterol to HDL ratio. A reduced ratio has been associated to improved cardiovascular health, reduced atherosclerotic risk, and decreased insulin resistance [51,52].

## Limitations

The population size should certainly be considered as a limitation in the current study. While the criteria for inclusion into the study were chosen to ensure that the participants were representative of the usual patient population undergoing obesity surgery, a population size of 30 is prone to skew by single outliers. This sample size particularly limits the statistical power for subgroup comparisons between LSG (*n* = 20) and RYGB (*n* = 11) procedures. The study may be underpowered to detect meaningful differences between surgical techniques. The absence of significant differences between LSG and RYGB in our study should therefore be interpreted with caution, as it may reflect insufficient power rather than true equivalence of outcomes. In cases of low statistical significance, results may be unique to the study or difficult to reproduce. However, the highly significant nature of the results discussed in the present study should provide some basis for the conclusions drawn in the discussion. As the findings were consistent in multiple evaluations, i.e., MRI volume, liver biomarkers, and clinical scoring system, and do not contradict each other, the findings can somewhat counteract the limited sample size.

Furthermore, The NAFLD fibrosis score has documented limitations in diabetic patients, where the presence of diabetes as both a score component and an independent fibrosis risk factor may reduce accuracy. The score relies on indirect biochemical and clinical parameters rather than direct histological or imaging assessment of fibrosis. In obesity surgery patients, rapid metabolic changes may alter score components (BMI, glycemic parameters, liver enzymes) independently of actual fibrosis changes. Alternative methods such as transient elastography or magnetic resonance elastography may provide more direct fibrosis assessment in future studies.

The absence of a control group represents an additional limitation. Without a non-surgical comparator group receiving standard medical management, we cannot directly assess the relative effectiveness of obesity surgery versus conservative treatment for MASLD. However, Patients meeting criteria for obesity surgery have established medical indications for surgical intervention, and randomizing such patients to non-surgical management for research purposes was not justifiable in the context of this prospective study. While this precludes direct comparison with conservative management in the current study, previous comparisons have shown efficacy of surgery compared to non-surgical intervention in larger study cohorts [53].

Finally, our study lacks histological confirmation of fibrosis improvement through liver biopsy, which remains the gold standard for assessing hepatic fibrosis. While ethical and practical considerations precluded routine biopsies in our patient population, this limits our ability to confirm that the biochemical improvements observed translate to actual histological regression of fibrosis.

## Conclusion

Obesity surgery led to significant reduction in liver volume, liver fat and pancreatic fat concentration, and improvement in MASLD and type 2 diabetes mellitus within the first year after surgery. Significant associations between visceral adipose, liver fat percentage, and NAFLD score were observed, highlighting the relationships between these factors in metabolic disease. These findings demonstrate that obesity surgery represents an effective treatment for MASLD and type 2 diabetes, with visceral obesity and total weight loss playing key roles in metabolic improvement. Future research should focus on long-term durability of metabolic improvements in larger patient cohorts, and the comparative effects of different surgical techniques to provide more tailored patient care.

## CRediT authorship contribution statement

**Hannes Götz Kenngott:** Project administration, Methodology, Conceptualization. **Philipp Anthony Wise:** Writing – original draft, Visualization, Validation, Investigation, Formal analysis. **Yixin Jiang:** Visualization, Formal analysis, Data curation. **Amila Cizmic:** Writing – review & editing, Investigation, Formal analysis, Data curation. **Felix Wagner:** Methodology, Investigation, Formal analysis, Data curation. **Hans-Ulrich Kauczor:** Resources, Project administration, Investigation, Conceptualization. **Adrian T. Billeter:** Writing – review & editing, Methodology, Formal analysis, Data curation. **Lars Fischer:** Methodology, Data curation, Conceptualization. **Johanna Nattenmüller:** Validation, Supervision, Project administration, Methodology, Investigation. **Beat Peter Müller-Stich:** Writing – review & editing, Supervision, Resources, Project administration, Investigation, Data curation, Conceptualization. **Rainer Grotelüschen:** Writing – review & editing, Validation, Supervision, Formal analysis. **Felix Nickel:** Writing – review & editing, Supervision, Project administration, Methodology, Investigation, Formal analysis, Conceptualization.

## Ethical approval

The POCCO study was approved by the ethics committee at Heidelberg University before inclusion of patients (S-450/2011). All procedures performed in studies involving human participants were in accordance with the ethical standards of the institutional and/or national research committee and with the 1964 Helsinki declaration and its later amendments or comparable ethical standards. All participants were briefed on the nature of the study and gave written informed consent.

## Funding/financial/acknowledgements

This work was supported by 10.13039/501100008334Stiftung Oskar-Helene-Heim.

## Declaration of competing interest

All authors have no related conflicts of interest to declare.
